# Exploring awareness and attitudes toward oocyte cryopreservation among women of reproductive age

**DOI:** 10.61622/rbgo/2025rbgo53

**Published:** 2025-07-15

**Authors:** Pedro Brandão, Rui Cândido, Fábio Carvalho, Liliane Nunes, Patrícia Matos, Pedro Frias, Marisa Coelho

**Affiliations:** 1 Universidade do Porto Porto Business School Porto Portugal Porto Business School, Universidade do Porto, Porto, Portugal.; 2 Next Fertility Portugal Faro Portugal Next Fertility Portugal, Faro, Portugal

**Keywords:** Cryopreservation, Fertility, Fertility preservation, Infertility, Oocytes, Women's health, Surveys and questionnaires

## Abstract

**Objective:**

This study aimed to evaluate awareness, perceptions, and attitudes toward oocyte cryopreservation among a diverse group of women.

**Methods:**

A cross-sectional study was conducted in November 2023 using snowball sampling to distribute a structured questionnaire. The target population included women aged 18+ with childbearing potential, proficient in English. An anonymous online survey with 22 questions collected data on demographics, education, occupation, relationship status, reproductive intentions, and perceptions of oocyte cryopreservation.

**Results:**

A total of 502 responses were analyzed. Most participants were in their thirties, had higher education, and were in committed relationships. They represented 24 countries, mainly in Europe. Over 60% planned to have children, but 85% were unfamiliar with oocyte cryopreservation. Social media (56%) was the primary information source, followed by acquaintances (33%) and healthcare professionals (25%). Only 4.6% were actively considering the procedure, while 41.3% showed potential interest. Barriers included perceived lack of necessity, age concerns, and limited information. Women considering cryopreservation were typically younger and childless. Financial constraints and information gaps significantly influenced decision-making, with about one-third suggesting better information and cost reduction could increase willingness to pursue the procedure.

**Conclusion:**

Despite relatively high awareness of oocyte cryopreservation, actual consideration and uptake remain low. Addressing financial and informational barriers could improve acceptance, especially among younger women and those uncertain about their reproductive plans.

## Introduction

Elective fertility preservation has become an increasingly relevant topic as more women choose to delay childbearing for personal and professional reasons. The decline in female fertility with age is well-documented, with significant decreases starting around age 35 and accelerating after age 37.^(1)^ This decline is due to the reduction in both the quantity and quality of oocytes. Women are born with approximately 1-2 million oocytes, but by age 51, only about 1,000 remain. Additionally, the risks of chromosomal abnormalities and pregnancy complications increase with maternal age, making conception more challenging and the outcomes less certain. There is approximately a 50% decrease in the fertility rate of women attempting pregnancy at age 40 or older compared with younger women, and a two to three-fold increased rate of spontaneous abortion. At 38 years old, about 50% of the resulting embryos are chromosomally abnormal.^(2–5)^

Despite these well-established biological facts, awareness about age-related fertility decline remains surprisingly low among both the general public and healthcare professionals. Several studies indicate a significant gap between knowledge about the optimal time frame for childbearing and the realistic chances of conception at older ages. For instance, research has shown that many women overestimate the effectiveness of assisted reproductive technologies (ART) in overcoming age-related fertility issues. In a survey of reproductive-aged women, only about half were aware that fertility begins to decline significantly in the mid-30s, and even fewer understood the steep decline after age 40.^(6–8)^

Parallel to the lack of awareness about natural fertility decline is the limited knowledge about oocyte cryopreservation as a method of elective fertility preservation. This procedure involves freezing and storing eggs at a younger age to use later when a woman is ready to conceive. Studies have demonstrated that even among healthcare professionals, knowledge about the details and success rates of oocyte cryopreservation is not widespread. For example, a survey among resident physicians across various specialties revealed that both ob-gyn and non-ob-gyn residents had a median score of just 2 out of 5 on fertility knowledge and 1 out of 3 on oocyte cryopreservation knowledge, indicating a substantial need for improved education in this area.^(9–11)^

The number of women opting for elective oocyte cryopreservation is growing, though it remains a relatively small proportion of the population. In certain demographics, particularly among well-educated, professional women in their mid-to-late 30s, the uptake is higher. Surveys show that while awareness of the option exists, actual engagement with the procedure is hindered by several factors. Some studies estimate that only about 33% of female university students are aware of the impact of age and knowledgeable about this kind of procedures, and only about 10% of young women have considered undergoing this procedure.^(12,13)^ Key barriers include the perception of the high cost of the procedure, lack of insurance coverage, and insufficient information provided by healthcare providers. Additionally, societal and personal factors such as career priorities and the absence of a suitable partner also play significant roles in the decision to delay childbearing and consider fertility preservation.^(14–17)^

Despite these barriers, the attitude towards elective fertility preservation is generally positive. Many women view oocyte cryopreservation as a means to take control of their reproductive future and reduce the anxiety associated with the ticking biological clock. Studies indicate that women who have undergone the procedure often feel a sense of relief and empowerment, even if they are aware that it does not guarantee a future pregnancy. As public awareness campaigns and educational initiatives improve, it is expected that more women will consider and opt for this technology, aligning their reproductive plans with their personal and professional aspirations.^(18–21)^

Although the decline in female fertility with age is an unavoidable biological phenomenon, elective oocyte cryopreservation provides a practical solution for women seeking to postpone childbearing. Nevertheless, there is an urgent need to improve awareness and comprehension of the limitations associated with natural fertility, as well as the advantages and limitations of fertility preservation technologies. By addressing these knowledge gaps and minimizing barriers to access, we can empower women to make informed choices regarding their reproductive health.

## Methods

This study was a cross-sectional analysis utilizing a snowball sampling method for questionnaire distribution. The initial participants were selected from the personal and professional contacts of the authors worldwide. These participants were encouraged to share the survey with other eligible women within their social and professional circles.

The study was conducted in November 2023. The target population comprised women over 18 years of age and of childbearing potential, with the inclusion criteria being women who could read and understand English and who consented to participate. Exclusion criteria were set for women under 18 years and those who did not consent.

Data collection was conducted via an anonymous online survey, distributed through platforms such as email, social media, and messaging applications. The survey was available exclusively in English and included 22 questions covering demographic information, educational background, occupational status, relationship status, reproductive intentions, and perceptions of oocyte cryopreservation.

The primary outcome of the study was to assess the level of awareness and understanding of oocyte cryopreservation among participants. Secondary outcomes included identifying barriers to opting for oocyte cryopreservation and analyzing factors influencing reproductive intentions.

Responses were collected confidentially via Google Forms and later transferred to a secure SPSS database for statistical analysis.

Descriptive statistics were used to summarize the dataset. Continuous variables were detailed through means and standard deviations, and categorical variables through frequencies and percentages. After assessing the normality of the continuous variables, parametric (T-test) and non-parametric (Mann-Whitney) tests were used to compare normally and non-normally distributed variables, respectively. The Chi-square test was used to compare categorical variables. Multivariate analysis was performed using logistic regression, excluding potential confounders, including age, which was significantly different between groups. We used a significance level of 0.05. Cases with missing data were excluded, on a per analysis basis.

## Results

We obtained 502 responses. The majority of the participants were in their thirties, had higher education, specifically a Bachelor's, Master's and Doctoral degree. Most were in a relationship, either married or cohabitating (63.5%), with 22% being single. About half already had children. The respondents were from 24 countries across five continents, with the majority from European countries (Table 1).

**Table 1:** Demographic characteristics of the study sample

Variables		n(%)
Age		
	<20	2(0.4)
	20-24	26(5.2)
	25-29	75(15.1)
	30-34	103(20.7)
	35-39	91(18.3)
	40-44	102(20.5)
	>45	99(19.9)
Level of studies		
	Elementary school	4(0.8)
	High school	28(5.6)
	Bachelor	114(22.7)
	Master	318(63.3)
	PhD	39(7.6)
Relationship status		
	Single	112(22.3)
	Married/Cohabiting	319(63.5)
	In a relationship, not married or cohabiting	69(13.7)
	Divorced / Widow	2(0.4)
Children		
	Yes	249(49.6)
	No	253(50.4)
Country of residence (ordered by number of participants)		Portugal, Belgium, the Netherlands, United Kingdom, Luxembourg, Mozambique, Spain, Switzerland, Brazil, Italy, United States of America, Austria, Bulgaria, Cape Verde, France, Ireland, Kenya, Norway, Perú, Qatar, Saudi Arabia, Russia and South Africa.

**Figure 1:**
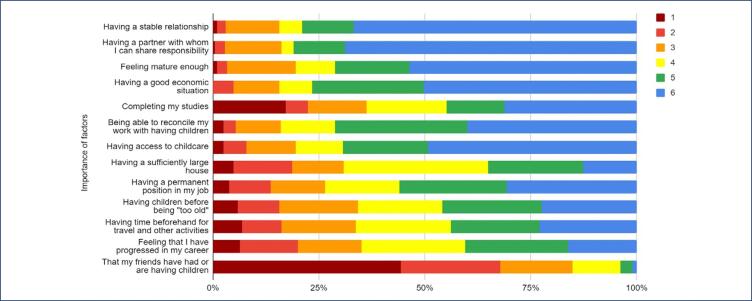
Key factors influencing respondents’ decisions on timing of motherhood (1 - least important, 6 - most important)

Over 60% of respondents refer plans to have children in the future and the majority place high importance on having children. The importance of various factors influencing the decision of when to become a mother varied among the respondents (Figure 1). Most women refer that "Having a stable relationship", "Having a partner with whom I can share responsibility", "feeling mature enough" and "having a good economic situation" and "completing my studies" are among the most important reasons to decide when to become mother.

**Figure 2:**
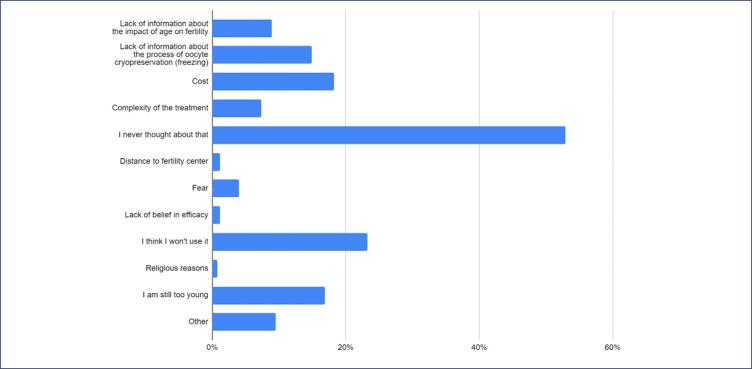
Factors contributing to the decision against oocyte cryopreservation

Approximately 13% considered themselves familiar with the procedure, 56% learned about it through social media, 33% through acquaintances, and 25% through healthcare professionals. About 80% knew that cryopreservation is a process of freezing eggs for preservation, 75% considered it an option for women who want to postpone motherhood, 69% believed it is used to maintain the viability of eggs for long periods, and 58% referred that it is an expensive procedure. Only 2 to 5% of respondents viewed oocyte cryopreservation as unhelpful in current clinical practice or expressed concerns that it might pose risks to the health of future children or the woman herself. More than 95% had not undergone oocyte cryopreservation, citing various reasons such as "I think I won't need it," "I am too young," and not being informed about the impact of age on fertility and the cryopreservation procedure. A total of 4.6% of respondents were considering the procedure and 41.3% said maybe. About 53% stated they do not consider it because "I never thought about that," 25% because "I think I won't use it," and around 15-18% referred that the reasons are due to lack of information, costs, and considering themselves too young for it (Figure 2).

**Figure 3:**
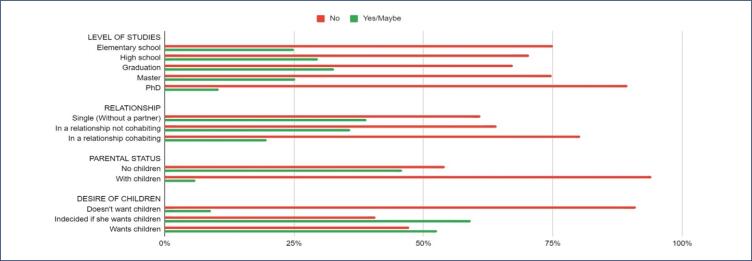
Percentage of participants considering oocyte cryopreservation, according to education level, relationship status, parental status, and future childbearing intentions

Compared to women who did not consider oocyte cryopreservation, those who said yes or maybe were younger (average age of 31 vs 40 years, p<0.01). Among women who already had children, only 6% responded yes or maybe to undergoing cryopreservation, while 46% of those without children considered the procedure (p<0.01). When comparing the possibility of undergoing the procedure considering the desire to become a mother, the percentage of women who responded yes or maybe was higher among those who were unsure if they wanted to become a mother in future (59%), compared to those who clearly wanted to be mothers (53%) or those who said they did not want children (9%) (p<0.01). No differences were observed at this level considering the level of education or relationship status (Figure 3).

**Figure 4:**
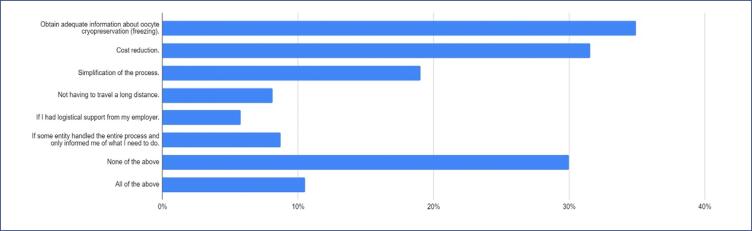
Factors that could positively influence the intention to undergo oocyte cryopreservation

About one-third of the participants believed that obtaining adequate information about oocyte cryopreservation and cost reduction could positively influence their decision to undergo cryopreservation (Figure 4).

**Figure 5:**
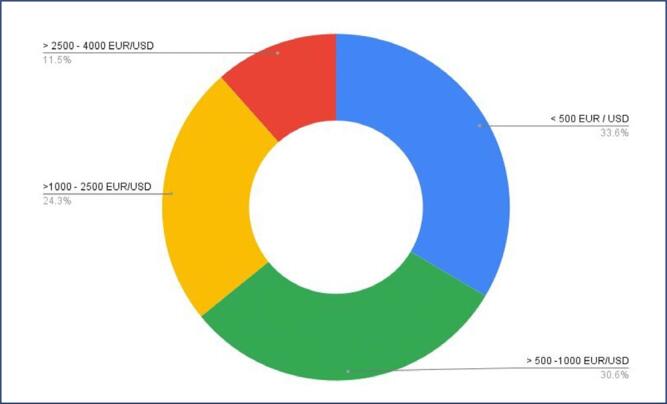
Participants’ responses on willingness to pay for oocyte cryopreservation

Almost two-thirds of the respondents would pay up to 500 or 1000 euros for the procedure (Figure 5).

## Discussion

Our study aimed to assess the awareness, perceptions, and attitudes towards oocyte cryopreservation among a diverse cohort of women. While most respondents were aware of the procedure, their levels of knowledge varied significantly. Some participants were moderately informed, while others had limited understanding, with only a small minority considering themselves well-versed in the process. These findings are consistent with previously published data.^(16,17,22)^

Only a small fraction of respondents (4.6%) were considering undergoing oocyte cryopreservation, but 41.3% indicated a possibility ("maybe"). Several barriers were identified, including perceived lack of necessity ("I think I won't need it"), age ("I am too young"), and insufficient information about the impact of age on fertility and the details of the cryopreservation process.^(21)^

The decision to consider cryopreservation was significantly influenced by the respondents’ age and parental status. Women who were considering or maybe considering the procedure were, on average, younger (31 years) compared to those who were not considering it (40 years). Furthermore, women without children were more likely to consider cryopreservation (46%) compared to those with children (6%). This underscores the perception of cryopreservation as a viable option primarily for younger women and those yet to start their families.

As expected, the desire to become a mother in the future also played a critical role. Women who were unsure about wanting to become mothers were more likely to consider cryopreservation (59%) compared to those who definitely wanted children (53%) or did not want children (9%). This suggests that cryopreservation may be perceived as a strategic option for preserving fertility in the face of uncertainty regarding future family planning.^(23)^

As stated in other studies, cost and information emerged as significant factors influencing the decision to undergo cryopreservation.^(24,25)^ A significant percentage of respondents revealed that they lacked information about the impact of age on female fertility and the cryopreservation procedure, which was an important factor for not having considered or undergone the procedure. About one-third of the respondents believed that obtaining adequate information and reducing the costs of the procedure could positively influence their decision. Almost two-thirds indicated a willingness to pay up to 500 or 1000 euros/US dollars for the procedure, highlighting the importance of making the procedure more accessible and affordable.

Interestingly, social media played a significant role in disseminating information about cryopreservation, cited by 56% of the respondents, followed by acquaintances (33%) and healthcare professionals (25%). This indicates the crucial role of digital platforms in raising awareness about fertility preservation options.^(21)^

This study has several limitations that should be acknowledged. Firstly, while the snowball sampling method enabled global reach, it may introduce selection bias, as participation depended on the engagement of the initial contacts and their networks. Additionally, since the survey was conducted exclusively in English, the sample may be biased toward participants with English proficiency. The high percentage of respondents with higher education could have biased the results, as this demographic might have greater interest and awareness regarding oocyte cryopreservation. However, it is also reflective of the population that may be most inclined to consider such procedures. Additionally, the questionnaire used in this study was not previously validated, which could impact the reliability of the data collected. The geographic representation of the respondents was not uniform, with a predominance of participants from high-development index countries, particularly in Europe. This uneven distribution may restrict the generalizability of the results to other regions with different socio-economic and healthcare contexts.

## Conclusion

Several barriers prevent a broader consideration and uptake of oocyte cryopreservation. Addressing these barriers through better information dissemination, cost reduction, and targeted education about the impact of age on fertility and the benefits and implications of cryopreservation could enhance its acceptance and utilization. Future research should explore strategies to bridge the gap between awareness and action, particularly focusing on younger women and those uncertain about their future reproductive plans.
